# Knee extensor muscle strength, physical function and patient-reported outcomes the first year after total knee arthroplasty: a prospective longitudinal study

**DOI:** 10.1186/s12891-025-09257-9

**Published:** 2025-10-21

**Authors:** Per Sjöström, Lena Nordeman, Ola Rolfson, Anette Larsson

**Affiliations:** 1 Primary Care Rehabilitation, Närhälsan Rehabilitation Centres, Lerum, Region Västra Götaland Sweden; 2 Department of Health and Rehabilitation, Institute of Neuroscience and Physiology, Sahlgrenska Academy, University of Gothenburg, Gothenburg, Sweden; 3 Region Västra Götaland, Research, Education, Development and Innovation, Primary Health Care, Södra Älvsborg, Borås, Sweden; 4 Department of Orthopaedics, Institute of clinical sciences, Sahlgrenska Academy, University of Gothenburg, Gothenburg, Sweden; 5 Orthopaedics, Sahlgrenska University Hospital, Gothenburg, Sweden; 6 General Practice/Family Medicine, School of Public Health and Community Medicine, Institute of Medicine, Sahlgrenska Academy, University of Gothenburg, Gothenburg, Sweden

**Keywords:** Total knee arthroplasty, Quadriceps muscle, Muscle strength, Physical therapy specialty, Muscle strength dynamometer, Primary health care, Patient reported outcome measures, Physical functional performance, Longitudinal studies

## Abstract

**Background:**

Knee extensor muscle strength is crucial for optimal knee function. Understanding the recovery process after total knee arthroplasty (TKA) and the impact of preoperative factors on recovery is therefore clinically significant. The primary aim was to investigate changes in knee extensor muscle strength in men and women during the first year after TKA. The secondary aim was to describe changes in physical function and patient-reported outcomes.

**Methods:**

A prospective observational study, with data collection before and at 12, 26 and 52 weeks after TKA. Participants (*n* = 57) were scheduled for elective primary TKA due to knee osteoarthritis. The primary outcome was changes in absolute and normalised maximum voluntary isometric contraction (AMVIC and NMVIC) for knee extensors in the operated knee, which was analysed with a linear-mixed effect model. Changes in physical function (6MWT and 30CST) were analysed with a paired samples t-test, and KOOS-scores were analysed with the Wilcoxon signed rank test.

**Results:**

The change in estimated marginal means for AMVIC at 12 weeks was − 27 N (95% CI -43;-11, *p* = .001) and − 3.1 percentage points for NMVIC (-5.7;-0.5, *p* = .013). At 52 weeks, the change was 42 N (21;63, *p* < .001) for AMVIC and 5.4 percentage points (2.1;8.6, *p* < .001) for NMVIC. The change at 26 weeks for AMVIC and NMVIC was not significant. AMVIC was affected by time, sex (both *p* < .001), statistical interaction between time and sex (*p* = .043) and the baseline covariates age (*p* = .010), BMI (*p* = .011) and 30CST (*p* = .048). NMVIC was affected by time, sex (both *p* < .001) and the baseline covariates BMI (*p* = .004), 6MWT (*p* = .023) and 30 CST (*p* = .036). 6MWT and KOOS-scores increased at all time points. 30CST increased from 26 weeks.

**Conclusions:**

Better preoperative physical function and lower BMI appear beneficial for postoperative recovery of normalised knee extensor muscle strength. Following TKA, patient-reported outcomes (symptoms, pain, ADL and knee-related QoL) and gait performance appear to improve more rapidly than knee extensor muscle strength and 30CST. Preoperative exercise and weight loss (if obese), as well as continued postoperative exercise even if pain is alleviated and walking is improved may be advisable.

**Trial registrations:**

Retrospectively registered 9 February 2022 (http://clinicaltrials.gov, ID: NCT05248815).

## Background

Total Knee Arthroplasty (TKA) is among the most common surgical procedures globally, with increasing numbers reported both worldwide and in Sweden [1, 2]. In Sweden, around 18,000 primary TKAs are performed annually, primarily for osteoarthritis [3]. Rehabilitation protocols following TKA aim to improve range of motion (ROM) and muscle strength in order to facilitate activities of daily living (ADL) [3]. Postoperatively, early rehabilitation in a primary care setting with guidance from a physiotherapist is recommended; however, there is no consensus regarding the optimal exercise strategy in terms of delivery, duration and intensity [3] since comparable improvements have been observed for pain, range of motion, function [4–7] and quality of life [7, 8]. Concerns have been raised about postoperative evaluation as it typically relies on range of motion tests and patient-reported outcomes but rarely includes objective tests of muscle strength and physical function [9, 10]. Therefore, relevant functional and activity related limitations may be missed [9, 10]. In addition, patient-reported function may correlate more to pain than to the actual performance on objective tests of physical function [11]. In Sweden, fast-track care programs have been broadly introduced [12] and all individuals are entitled to access physiotherapy services as part of the publicly funded primary healthcare system. Most patients are satisfied after primary TKA, however, approximately 8% report overall dissatisfaction with surgery after one year [13]. It has been suggested that the primary mechanism behind dissatisfaction is unmet expectations, and patients have expressed the need for more accurate information regarding recovery (e.g. knee function, ADL and pain reduction/cessation) during the first year, rather than simply being informed that recovery may take up to a full year [14]. This implies that there is a need for information about the recovery process for different time points during the first year, as well as information based on both objective and subjective outcome measures.

Knee extensor muscle strength is fundamental for optimal knee function and is highly related to functional performance following Total Knee Arthroplasty (TKA) [15–17]. Knee extensor muscle strength declines as age increases [18, 19] and is also sex [20] and body-size dependent [20, 21]. Normalised knee extensor muscle strength (in relation to body weight) is recommended over absolute strength for comparison between groups or individuals [21] and shows better correlation with tests of physical function following TKA [15]. Patients undergoing TKA due to osteoarthritis (OA) generally have lower knee extensor muscle strength compared to age-matched controls [22, 23] and muscle strength is weaker on the operated side compared to the contralateral side before and after surgery [24]. After TKA, knee extensor muscle strength on the operated side initially decreases [25, 26] and then gradually increases from 3 to 6 months and up to 2 years after surgery [26]. The association between sex, age, and BMI on knee extensor muscle strength recovery after TKA have previously been studied with inconclusive results and more studies are needed [26]. Therefore, this study will further investigate how these individual factors are associated with knee extensor muscle strength following TKA, alongside objective measures of physical function. The results may be valuable for patients and healthcare professionals in pre- and postoperative evaluation, planning and communication processes.

In summary, knowledge about changes in knee extensor muscle strength, physical function and patient-reported outcome measures during the first year following TKA is crucial for both patients and health-care professionals. However, there is limited knowledge about how preoperative factors are associated with changes in knee extensor muscle strength. Therefore, we conducted this study with the primary aim of investigating changes in knee extensor muscle strength in men and women during the first year after TKA. The secondary aim was to describe changes in physical function and patient-reported outcomes.

## Methods

### Study design

A prospective observational study was conducted with repeated measurements up to 52 weeks after surgery. Testing was performed at three primary care facilities at four time points: baseline (0–2 weeks before surgery) and at 12, 26 and 52 weeks after surgery. The study was approved by the Swedish Ethical Review Authority, Sweden (DNR 2021–03590 and DNR2024-04643-02). All study methods were performed in accordance with the Declaration of Helsinki. This study is retrospectively registered in the Clinical Trials Registry (ID NCT05248815).

### Participants

Participants were scheduled to undergo elective primary TKA due to knee OA at Alingsås Hospital (Alingsås, Sweden) and were asked to participate after oral and written information provided by the nurse during preoperative information. Alingsås Hopsital uses fast-track care as defined in a previous study [12]. Those willing to participate were then scheduled for baseline testing. The inclusion criteria were as follows: 65 years of age or older, elective primary TKA due to knee OA and expected to participate in primary care rehabilitation. Exclusion criteria were inability to understand, read or speak Swedish. All participants received the same guideline-based standard care rehabilitation, consisting of a standardized postoperative exercise program (range of motion exercises and quadriceps activations performed three times daily) and follow-up appointments with a primary care physiotherapist at 1, 3, and 6 weeks postoperatively. Progression of the exercise program occurred at 3 weeks (with the addition of weight-bearing exercises and calf muscle stretching) and at 6 weeks (with the inclusion of step-ups, balance training, and resistance band exercises). Range of motion exercises were continued as needed. From 6 weeks onward, rehabilitation was individually tailored, allowing participants to attend weekly exercise sessions at the physiotherapy department or to continue independent training based on given instructions, with or without follow-up.

**Fig. 1:**
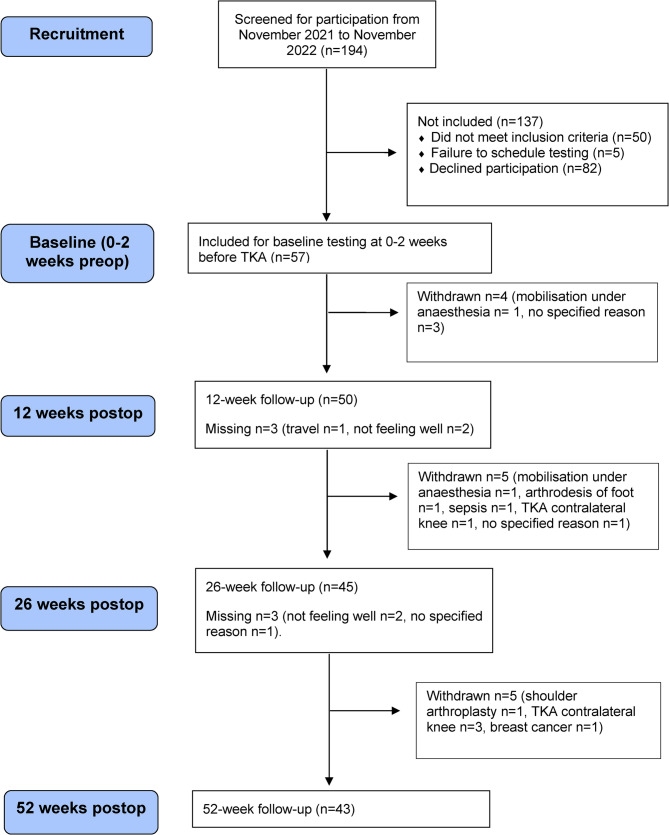
Study flow-chart. Missing: Number of participants not attending follow-up and reasons for not attending. Participants still in the study and scheduled for next follow-up. Withdrawn: Number of participants withdrawn and reasons for withdrawal, no more follow-ups. TKA: Total Knee Arthroplasty

The principal investigator (PS) was not involved in the rehabilitation of any of the participants. One hundred and ninety-four participants were eligible for inclusion, 82 declined, 50 did not meet inclusion criteria and 5 were excluded due to the failure to schedule preoperative testing. Finally, 57 participants were included. Fourteen participants did not complete 52-week follow-up, 8 of them were men. At postoperative testing, participants were withdrawn from the study if events occurred that were considered likely to impact recovery and test results. These events included complications following TKA (mobilisation under anaesthesia *n* = 2) or other medical conditions, treatments or surgeries (e.g. additional joint arthroplasty *n* = 5, cancer diagnosis and treatment *n* = 1, arthrodesis of foot *n* = 1, sepsis *n* = 1). The remaining four withdrew without specified reason. The study flowchart is presented in Fig. 1.

### Data collection

Data were collected from November 2021 to November 2023. Testing and data collection was performed at three different primary care facilities in Region Västra Götaland, Sweden. Participant baseline characteristics collected to describe the study group were age, sex, height, weight, Body Mass Index (BMI) as calculated from height and weight, and previous joint arthroplasty in hip and/or knee. Baseline testing was performed 0 to 2 weeks before surgery. Postoperative testing was scheduled at 12 weeks, 26 weeks and 52 weeks after surgery. At all time points, data were collected for knee extensor muscle strength, 30 s Chair Stand Test (30CST), 6-min Walk Test (6MWT), Knee injury and Osteoarthritis Outcome Score (KOOS) and physical activity level. Overall satisfaction was rated 52 weeks after surgery.

### Knee extensor muscle strength and physical function

Knee extensor muscle strength was measured as maximum voluntary isometric contraction (MVIC) using a make test with a fixated MicroFET2 handheld dynamometer (Hoggan Scientific, Salt Lake City, UT, USA). After TKA, testing with fixation has been shown to have excellent inter-rater reliability, high validity and is recommended over testing without fixation [27]. Intra-rater reliability without fixation is excellent [20] and is assumed to be similar with fixation. It is recommended that the performance of each test lasts five seconds in order to achieve MVIC [20] and that the result is expressed in relation to body weight [21]. Data were obtained with the dynamometer fixated against the lower leg lever in a leg extension apparatus (Leg extension 4000, Ergo Fit, Pirmasens, Germany), with knees at 90 degree flexion, arms folded across the chest and back against the back rest. Participants were asked to exert maximum force for five seconds while receiving verbal encouragement. First, the operated side was tested, followed by 60 s of rest. Next, testing was performed on the contralateral side. The procedure was repeated until up to three tests were performed on both sides. The best result from both sides was recorded as absolute maximum voluntary isometric contraction (AMVIC), expressed in Newtons (N), and as normalised maximum voluntary isometric contraction (NMVIC), expressed as a percentage of body weight given by the formula AMVIC/body weight * 100 (AMVIC and body weight in N).

Physical function was measured with 6MWT and 30CST. 6MWT is a generic test used to evaluate physical function, where walking distance (m) in six minutes is recorded. The test is widely used for various conditions, including TKA [28, 29]. 6MWT demonstrates good reliability [29, 30] and sensitivity to change [29, 30] following TKA. The test was performed according to guidelines from the Osteoarthritis Research Society International (OARSI) [31]. A 20 m long corridor was used.

30CST measures the number of stand ups (n) in 30 s from a chair (height 44 cm) with arms folded across the chest [31, 32]. Testing was performed according to OARSI guidelines [31]. The test is valid as a measure of lower extremity physical function for people aged 60–89 years [33], reliability is excellent [34] and its use is recommended by OARSI for both research and clinical purposes following TKA [31].

### Patient-reported outcome measures

KOOS is a 42-item patient-reported outcome measure (PROM) divided into five dimensions: pain (KOOS-pain, 9 questions), other symptoms (KOOS-symptoms, 7 questions), ADL (KOOS-ADL, 17 questions), sport and recreation (KOOS-sport, 5 questions) and knee-related quality of life (QoL) (KOOS-QoL, 4 questions). Each question has five possible answers (e.g. none, mild, moderate, severe, extreme), which are graded 0–4. For each subscale, a total score from 0 to 100 is calculated, where 0 equals extreme difficulties and 100 no difficulties. Differences in KOOS-subscores and changes over time will be visualised by plotting KOOS-profiles as recommended from the user’s guide [35]. KOOS-sport was not used as it consists of questions about activities such as running and jumping, which were not relevant to the scope of this study. Reliability [36] and validity [37] following TKA is good. The 12-item version is used in the Swedish Arthroplasty Register [13].

Physical activity level was obtained using patient-reported measurement as recommended by the Swedish National Board of Health and Welfare [38] and was classified as sufficient (≥ 150 min/week) or insufficient (< 150 min/week).

For overall satisfaction one year after surgery, a 4-point Likert scale was used (very satisfied, somewhat satisfied, somewhat dissatisfied, very dissatisfied). The use of a Likert scale is the most common method to evaluate overall satisfaction following TKA [39].

### Statistical analysis

Baseline characteristics are reported as mean (standard deviation (SD)), median (min-max) and number (%). Continuous data were assessed for normality with the visual inspection of a histogram, boxplots and normality plots, Kolmogorov-Smirnov and test for skewness. Based on this assessment, a paired samples t-test was used to analyse within group differences over time. For KOOS, Wilcoxon-signed rank test was used to analyse within group differences over time.

Linear mixed effect model analysis for repeated measures was used to further investigate changes in AMVIC and NMVIC on the operated side for men and women over 12 months, with adjustment for potential baseline covariates. Independent variables in the model were checked for collinearity using Spearman’s rank correlation (*r* ≤.7) and visual inspection of scatterplots [40]. The mixed effect model analysis of AMVIC on the operated side consisted of two models: Model 1 and Model 2. In both models, random intercepts were included for each patient: Baseline (0–2 weeks preop) and female sex were treated as reference values when comparing estimates of fixed effects. Model 1 (unadjusted): Variables: time, sex and time*sex (interaction between time and sex). Model 2 (final model with covariate adjustments): Based on model 1 with covariate adjustments as described below. Potential covariates were baseline age, BMI, 6MWT and 30CST and were added one at a time to Model 1 and carried forward to the final model if *p* <.2. Variables in the final model (time, sex and time*sex) were considered statistically significant if *p* <.05. Mixed effect model analysis of NMVIC on the operated side followed the same procedure as described above. Estimates for significant baseline covariates in Model 2 will be presented with a 95% CI. The model estimated marginal means will be in graph form to illustrate changes in AMVIC and NMVIC for women and men over 12 months. Bonferroni adjustments for multiple comparisons were done for pairwise comparisons of estimated marginal means. Post hoc analyses performed to test for attrition bias were: (1) sensitivity analysis by conducting linear mixed effect model analyses as in our final model for AMVIC and NMVIC, but only in participants with complete baseline and 52-week data; (2) between group analyses of baseline characteristics and test results between those with complete baseline and 52-week follow-up and those with incomplete data (*n* = 18), which were performed with Mann-Whitney U Test for continuous variables and chi-square test for dichotomous variables.

A sample size of 22 subjects was required to detect a 50 N change in knee extensor maximal voluntary isometric contraction (MVIC), based on a power calculation utilising a paired samples t-test. This calculation assumed a 5 per cent significance level, 80 per cent power and a standard deviation (SD) of 80 N. Statistical tests were performed using the Statistical Package for Social Sciences version 28.0 (SPPS, Inc., Chicago, IL). Statistical significance was set at *p* <.05.

## Results

### Study group and baseline data

Baseline characteristics are presented in Table 1. Participants (*n* = 57) had a mean age of 73 (SD 4,9) years, a majority were men (61%), mean BMI was 28.1 (SD 3.3), a majority were classified as sufficiently physically active (74% >150 activity minutes per week), a little more than half were scheduled for TKA on the right knee and about a third (33%) had previously been operated with joint arthroplasty in one hip/both hips and/or on the contralateral knee. Fifty (88%), 44 (77%) and 43 (75%) participants attended the 12-week, 26-week and 52-week follow-up, respectively. Thirty-five participants (81%) were very satisfied, 7 participants (16%) somewhat satisfied and 1 participant (2%) was somewhat dissatisfied when rating overall satisfaction 52 weeks after surgery.

**Table 1:** Baseline characteristics (n=57)

	n (%)
Sex (women)	22 (39)
	Mean (SD) Median (min;max)
Age (years)	73.3 (4.9)
	74.0 (65;85)
Height (cm)	173.1 (9.3)
	174.0 (145;188)
Weight (kg)	84.5 (14.9)
	81.0 (53;125)
BMI (kg/m^2^)	28.1 (3.3)
	28.1 (22.0;35.6)
	n (%)
Operated side	
Left	25 (44)
Previous arthroplasty	
No	39 (68)
Right hip only	2 (4)
Left hip only	0 (0)
Both hips, no knee	3 (5)
Contralateral knee only	11 (19)
Contralateral knee+ left hip	1 (2)
Contralateral knee+ right hip	1 (2)
Sufficient physical activity (>150 min/week)	
Yes	42 (74)

*BMI* Body Mass Index, *SD* Standard deviation

### Physical function (AMVIC, NMVIC, 6MWT and 30CST)

AMVIC and NMVIC in the operated knee decreased from baseline to 12-week follow-up (*p* =.002 and 0.004) and was increased at 26- and 52-week (*p* <.01) follow-up, with the biggest increase between the 12- and 26-week follow-up.

AMVIC in the contralateral knee increased from baseline to the 26- and 52-week follow up (both *p* <.001) and from the 12-week follow up to the 26-week (*p* =.007) and 52-week follow up (*p* =.007). Similarly, NMVIC in the contralateral knee increased from baseline to the 26-week and 52-week follow up (both *p* <.001) and from the 12-week follow up to the 26-week (*p* =.004) and 52-week follow up (*p* =.005).

6MWT increased from baseline to the 12-week follow-up (*p* =.008) and between all other time points (*p* <.001). 30CST increased from 12 weeks to 26 weeks (*p* <.001) and from 26 weeks to 52 weeks (*p* <.001). Baseline test results and changes in knee extensor muscle strength and physical function are presented in Table 2.

**Table 2:** Baseline data and changes in knee extensor muscle strength and physical function

	T0 (*n* = 56)		ΔT0-T1 (*n* = 47)		ΔT0-T2 (*n* = 40)		ΔT0-T3 (*n* = 39)		ΔT1-T2 (*n* = 39)		ΔT1-T3 (*n* = 38)		ΔT2-T3 (*n* = 37)	
	Mean (SD)	Median (min; max)	Mean (SD) 95% CI	*p*-value	Mean (SD) 95% CI	*p*-value	Mean (SD) 95% CI	*p*-value	Mean (SD) 95% CI	*p*-value	Mean (SD) 95% CI	*p*-value	Mean (SD) 95% CI	*p*-value
AMVIC operated knee (N)	311 (102)	305 (131;542)	−25 (53) −40; −9	**0.002**	29 (61) 9;48	**0.005**	68 (58) 49; 87	**< 0.001**	63 (48) 47; 78	**< 0.001**	93 (56) 74; 111	**< 0.001**	33 (30) 23; 44	**< 0.001**
NMVIC operated knee (%)	37.3 (9.3)	36.6 (16.3;58.0)	−2.7 (6.1) −4.5;−0.9)	**0.004**	3.8 (7.0) 1.6; 6.1)	**< 0.001**	8.5 (6.9) 6.2; 10.7	**< 0.001**	7.5 (5.1) 5.8; 9.2	**< 0.001**	11.2 (6.2) 9.1; 13.2	**< 0.001**	3.9 (3.6) 2.7; 5.1	**< 0.001**
AMVIC contralateral knee (N)	355 (115)	333 (184;632)	11 (44) −1.8; 24	0.090	26 (46) 11; 40	**< 0.001**	37 (47) 21; 53	**< 0.001** ‡	15 (33) 4;26	**0.007**	22 (44) 6;37	**0.007** **§**	10 (33) −1.5; 22	0.086 §§
NMVIC contralateral knee (%)	43.0 (12.4)	42.0 (22.5;82.7)	1.3 (5.3) −0.3; 2.8	0.105	3.2 (5.3) 1.5; 4.9	**< 0.001**	4.5 (6.0) 2.4; 6.5	**< 0.001** ‡	2.0 (4.1) 0.7; 3.4	**0.004**	2.9 (5.7) 0.9; 4.9	**0.005** **§**	1.2 (4.2) −0.3; 2.7	0.103 §§
6MWT (m)	406 (108)	409 (180;740)	27 (65) 7;47	**0.008** ††	61 (49) 46; 77	**< 0.001**	83 (55) 64; 100	**< 0.001** ‡‡	39 (42) 25; 53	**< 0.001** ‡‡‡	57 (46) 41; 72	**< 0.001** ‡‡	23 (24) 14; 31	**< 0.001** **§**
30CST (n)	9.1 (3.9) †	10 (0;22) †	0.7 (3.2) −0.3; 1.7	0.145	2.5 (3.5) 1.5; 3.6	**< 0.001** †††	4.1 (2.9) 3.1; 5.0	**< 0.001**	1.8 (3.2) 0.7; 2.8	**0.001**	3.1 (2.9) 2.1; 4.1	**< 0.001** ‡‡‡	1.6 (3.0) 0.6; 2.6	**0.003** ‡‡‡

*6MWT* 6-Min Walk Test, *30CST* 30 s Chair Stand Test, *AMVIC* Absolute Maximum Voluntary Isometric Contraction, *NMVIC* Normalised Maximum Voluntary Isometric Contraction = strength as % of body weight, *SD* Standard deviation, *T0* Baseline, 0–2 weeks before surgery, *T1* 12-week follow-up, *T2* 26-week follow-up, *T3* 52-week follow-up

Presenting baseline test results and differences between different time points with 95% CI, as well as p-values for the paired samples t-test (significant changes in bold). †: n = 57. ††: n = 45. †††: n = 43. ‡: n = 35. ‡‡: n = 38. ‡‡‡: n = 36. §: n = 34. §§: n = 33

### Patient-reported outcomes (KOOS)

**Fig. 2:**
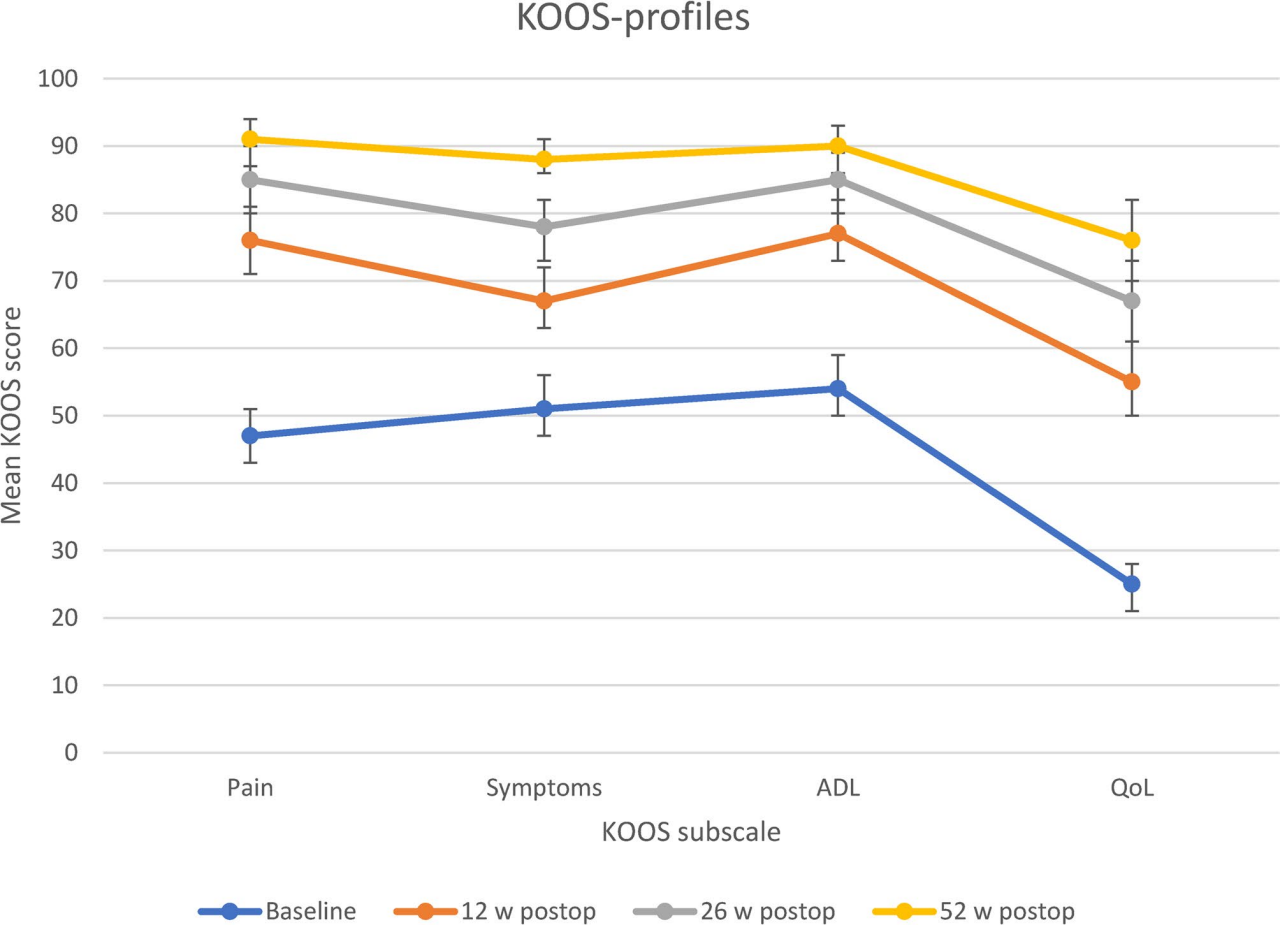
KOOS profiles from baseline (0–2 weeks before TKA) to 52-weeks after TKA. Mean KOOS scores with 95% CI at baseline (0–2 weeks before TKA, n=57), 12 weeks (n=50), 26 weeks (n=44) and 52 weeks (n=43) after TKA. ADL: Activities of daily living. CI: Confidence interval. KOOS: Knee osteoarthritis outcome score. TKA: Total knee arthroplasty. QoL: Quality of life

Mean KOOS-scores increased at all time points for all subscales. All changes were statistically significant (*p* <.001). The greatest increase for all subscales was from baseline to 12 weeks postoperative. Changes in KOOS profiles are illustrated in Fig. 2.

### Linear Mixed effect model analysis of AMVIC in the operated side

All 57 participants were included in the mixed effect model analysis for AMVIC. All potential baseline covariates in Model 1 (age, BMI, 6MWT and 30CST) were carried forward to Model 2 (all *p* <.2). In Model 2 (final), for AMVIC in the operated knee, time, sex and the statistical interaction between time and sex were all statistically significant (*p* <.05). Additionally, age, BMI and 30CST were identified as significant baseline covariates (*p* <.05). Male sex, higher BMI and better 30CST performance were associated with higher values for the AMVIC in the operated knee. Age was linked to lower values. 6MWT was not a significant baseline covariate. See Table 3 for results from the linear mixed effect model 2 (final).

**Table 3:** Mixed effect model analysis of AMVIC from baseline to 52-week follow-up

	Model 2 (final model)		Sensitivity analysis
Variables	Estimates (95%CI)	*p*-values	*p*-values
Time	**-**	**< 0.001**	**< 0.001**
Baseline preop	*	-	-
12 w postop	-17.1 (-41.5;-7.4)	0.166	0.094
26 w postop	21.2 (-7.0;49.3)	0.138	0.298
52 w postop	**31.5 (1.4;61.5)**	**0.041**	0.172
Sex	139.9 (104.2;175.6) **	**< 0.001**	**< 0.001**
Time x Sex	**-**	**0.045**﻿	0.059
Baseline preop x Sex	*	-	-
12 w postop x Sex	-20.6 (-51.8;10.6)	0.191﻿	0.528
26 w postop x Sex	-8.5 (-43.7;26.7)	0.630	0.867
52 w postop x Sex	21.4 (-14.6;57.4)	0.238	0.086
Baseline covariates (fixed data)			
Age	**−4.0 (−7.0;−1.0) **	**0.010**	0.201
BMI	**5.8 (1.4;10.2)**	**0.011**	**0.005**
6MWT	0.15 (−0.012;0.30)	0.071	0.056
30CST	**2.9 (0.03;5.8)**	**0.048**	**0.007**

Presenting *p*-values for Model 2 (final model, *n* = 57) and sensitivity analysis (*n* = 39) from regression analyses using linear mixed effect model and estimates with 95% confidence interval for Model 2, significant estimates in bold. All potential covariates analysed in Model 1 (all *p* <.2) were carried forward to Model 2

Sensitivity analysis: Linear mixed effect model for participants with complete baseline and 52-week follow-up (*n* = 39), same variables as Model 2. Time x sex: Statistical interaction between time and sex. Baseline and female were used as model reference values

*6MWT* 6-min walk test, *30CST* 30 s chair stand test *AMVIC* Absolute maximum voluntary isometric contraction, *BMI* Body Mass Index, *CI* Confidence interval

* Model reference value

**Estimate for males

**Fig. 3:**
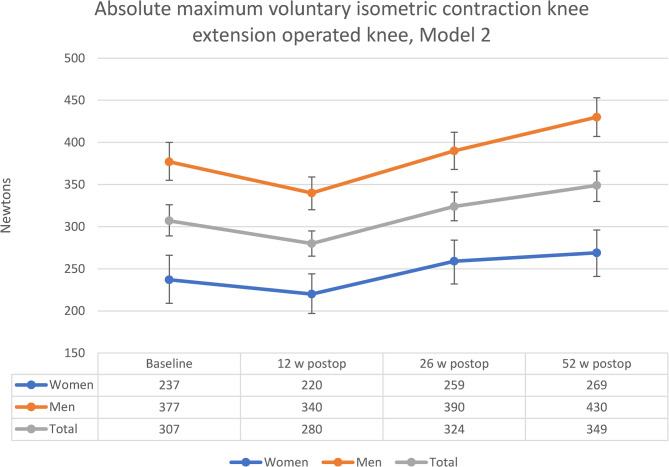
Estimated marginal means and 95% CI for absolute maximum voluntary isometric contraction knee extension in operated knee for women, men and total group. Model 2 adjusted for potential covariates: Age = 73.3432, body mass index = 27.8549, 6-min walk test = 449.2308, Chair Stand Test 30 s = 11.0355

The final model estimated marginal means for AMVIC from baseline to the 12-week follow-up was − 27 N (−43;−11, *p* =.001), 17 N (−2.3;36, *p* =.083) to the 26-week follow-up and 42 N (21;63, *p* <.001) to 52-week follow-up. The final model estimated marginal means for AMVIC decreased from baseline to the 12-week follow-up for both men (−37 N or −9.8%) and women (−17 N or −7.2%). The greatest increase was from the 12-week follow-up to the 26-week follow-up for both men (50 N or 14.7%) and women (39 N or 17.7%). At the 26-week follow-up, AMVIC exceeded baseline results for both men (13 N or 3.4%) and women (22 N or 9.3%). AMVIC continued to increase from the 26-week follow-up to the 52-week follow-up for both men (40 N or 10.3%) and women (10 N or 3.9%). Total difference from baseline to the 52-week follow-up for men was 53 N (14.1%) and for women 32 N (13.5%). For men, baseline estimated marginal mean was 377 N (95% CI 355;400) and 430 N (95% CI 407:453) at the 52-week follow-up. For women, baseline estimated marginal mean was 237 N (95% CI 209;266) and 269 N (95% CI 241;296) at the 52-week follow-up. Changes in final model estimated marginal means are illustrated in Fig. 3.

### Linear Mixed effect model analysis of NMVIC operated side

All 57 participants were included in the mixed effect model analysis for NMVIC. All potential baseline covariates in Model 1 (age, BMI, 6MWT and 30CST) were carried forward to Model 2 (all *p* <.2). In Model 2 (final), for NMVIC in the operated knee, time and sex were significant (both *p* <.001). Additionally, BMI (*p* =.004), 6MWT (*p* =.023) and 30CST (*p* =.036) were identified as significant baseline covariates. Male sex, lower BMI and better 6MWT and 30CST performance were associated with higher values for NMVIC in the operated knee. The statistical interaction between time and sex, as well as age, was not significant. See Table 4 for results from the linear mixed effect model 2 (final).

**Table 4:** Mixed effect model analysis of NMVIC from baseline to 52-week follow-up

	Model 2 (final model)		Sensitivity analysis
Variables	Estimates (95%CI)	*p*-values	*p*-values
Time Baseline preop 12 w postop 26 w postop 52 w postop Sex Time x Sex Baseline preop x Sex 12 w postop x Sex 26 w postop x Sex 52 w postop x Sex	**- ** * −2.2 (−5.1;0.8) **3.5 (0.2;6.7)** **5.1 (1.6;8.5)** 11.2 (7.0;15.4) ** **-** * −1.8 (−5.6;1.9) −2.2 (−6.3;1.8) 0.6 (−3.5;4.7)	**< 0.001** - 0.142 **0.037** **0.005** **< 0.001** 0.344 - 0.326 0.273 0.780	**< 0.001** - 0.087 0.128 **0.045** **< 0.001** 0.444 - 0.741 0.710 0.371
Baseline covariates (fixed data)			
Age	− 0.2 (−0.6;−0.1)	0.225	0.981
BMI	**− 0.8 (−1.3;−0.3)**	**0.004**	**0.032**
6MWT	**0.022 (0.003;0.041)**	**0.041**	**0.010**
30CST	**0.37 (0.03;0.70)**	**0.036**	**0.009**

Sensitivity analysis: Linear mixed effect model for participants with complete baseline and 52-week follow-up (*n* = 39), same variables as Model 2. Time x sex: Statistical interaction between time and sex. Baseline and female were used as model reference values

Presenting *p*-values for Model 2 (final model, *n* = 57) and sensitivity analysis (*n* = 39) from regression analyses using linear mixed effect model and estimates with 95% confidence interval for Model 2, significant estimates in bold. All potential covariates analysed in Model 1 (all *p* <.2) were carried forward to Model 2

*6MWT* 6-min walk test, *30CST* 30 s chair stand test, *NMVIC* Normalised maximum voluntary isometric contraction (% of body weight), *BMI* Body Mass Index, *CI* Confidence interval

* Model reference value

**Estimate for males

**Fig. 4:**
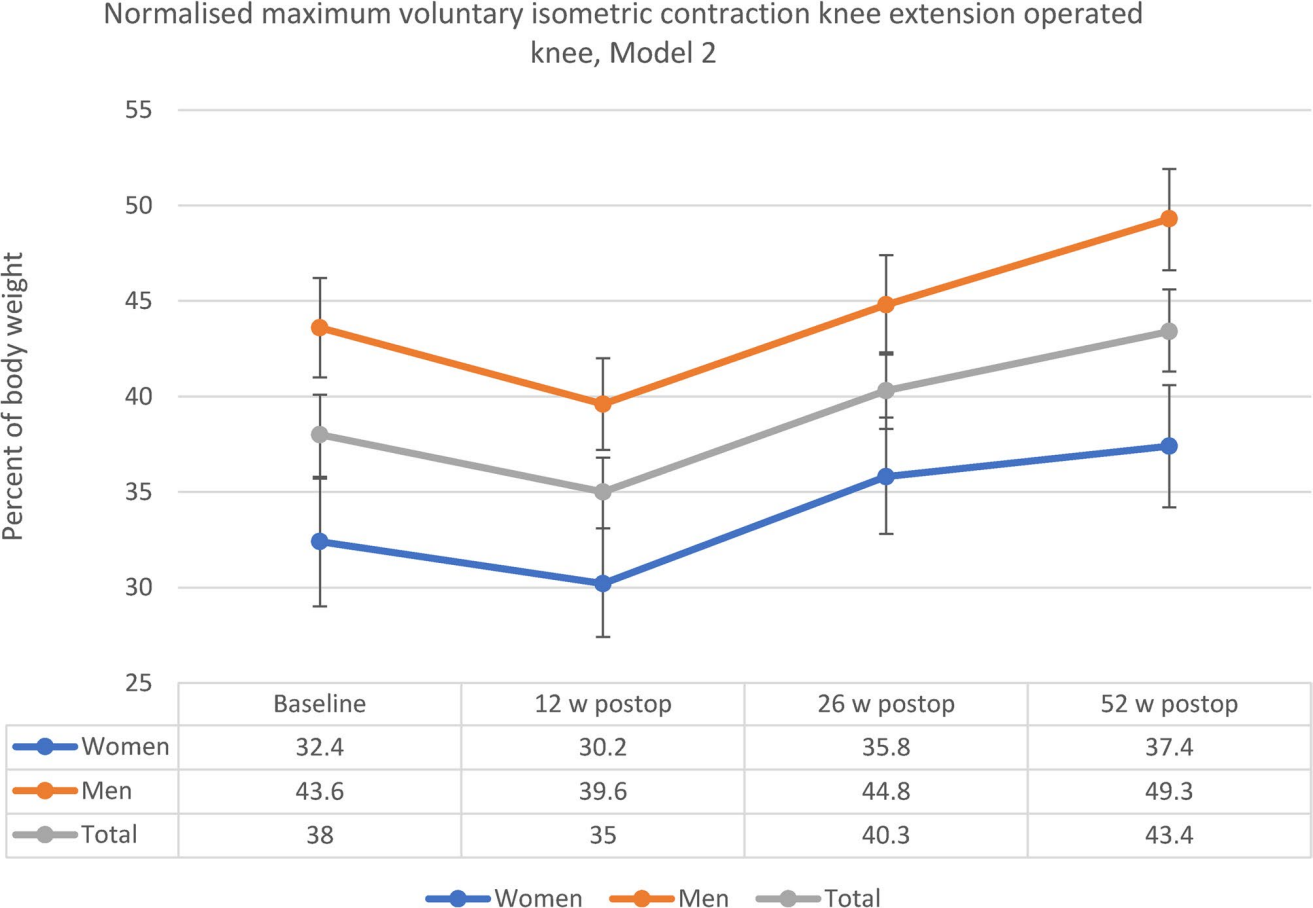
Estimated marginal means for normalised maximum voluntary isometric contraction knee extension in operated knee for women, men and total group. Model 2 adjusted for potential covariates: Age = 73.3432, Body Mass Index = 27.8549, 6-min walk test = 449.2308, Chair Stand Test 30 s = 11.0355

The final model estimated marginal means for NMVIC from baseline to the 12-week follow-up was − 3.1 percentage points (−5.7;−0.5, *p* =.013), 2.3 percentage points (−0.7;5.3, *p* =.236) to the 26-week follow-up and 5.4 percentage points (2.1;8.6, *p* <.001) to the 52-week follow-up. The final model estimated marginal means for NMVIC decreased from baseline to the 12-week follow-up for both men (−4.0 percentage points or −9.2%) and women (−2.2 percentage points or −6.8%). The greatest increase was from the 12-week follow-up to 26-week follow-up for both men (5.2 percentage points or 13.1%) and women (5.6 percentage points or 18.5%). At the 26-week follow-up, NMVIC exceeded baseline results for both men (1.2 percentage points or 2.8%) and women (3.4 percentage points or 10.5%). NMVIC continued to increase from the 26-week follow-up to the 52-week follow-up for both men (4.5 percentage points or 10.0%) and women (1.6 percentage points or 4.5%). Total difference from baseline to the 52-week follow-up for men was 5.7 percentage points (13.1%) and for women, 5 percentage points (15.4%). For men, baseline estimated marginal mean for NMVIC was 43.6% (95% CI 41;46.2) and at the 52-week follow-up, 49.3% (95% CI 46.7;51.9). For women, the baseline estimated marginal mean for NMVIC was 32.4% (95% CI 29;35.7) and 37.4% (95% CI 34.2;40.6) at the 52-week follow-up. Changes in NMVIC estimated marginal means are illustrated in Fig. 4.

### Post hoc analysis

Post hoc linear mixed-effect model analysis (sensitivity analysis) for participants with complete baseline and 52-week data (*n* = 39) for AMVIC (see Table 3) differed from our final model as statistical interaction between time and sex (*p* =.059 from *p* =.045) and age were no longer significant (*p* =.201 from *p* =.010). For NMVIC (see Table 4), results were similar as in our final model for overall effect of time, sex, statistical interaction between time and sex and baseline covariates age, BMI, 6MWT and 30CST. For baseline data, participants without a 52-week follow-up (*n* = 18) had significantly lower KOOS-pain (*p* =.018), KOOS-ADL (*p* =.015) and 30CST performance (*p* =.002) compared to participants with complete baseline data and 52-week data (*n* = 39).

## Discussion

### Summary of findings

This study investigates changes in knee extensor muscle strength in the operated knee for men and women during the first year after TKA and describes changes in physical function and patient-reported outcomes. The findings of this study indicate that knee extensor muscle strength in the operated knee initially decreases, followed by an increase from the 12-week follow-up. Changes in absolute and normalised knee extensor muscle strength may both be affected by time after surgery and, to a varying degree, preoperative factors such as age, sex, BMI and physical function. The time span for improvements in physical function appear to vary between different tests. Patient-reported pain, other symptoms, ADL and knee-related QoL all improved most in the initial period after surgery and thus appear to improve faster than lower extremity physical function.

### AMVIC in the operated knee

Consistent with findings in two meta-analyses [24, 26] we found a significant decrease from baseline to the 12-week follow-up, no significant changes to the 26-week follow-up and a significant increase to the 52-week follow-up for both AMVIC and NMVIC. Similar to our findings, higher BMI, male sex and lower age have been associated with higher AMVIC in previous research in healthy individuals [19–21, 41]. Consistent with our findings following TKA, a meta-analysis including both AMVIC and NMVIC [24] found that a higher proportion of female participants were negatively correlated with changes in knee extensor muscle strength in the operated knee. In contrast to our findings, the same meta-analysis [24] found that BMI was negatively correlated with changes in knee extensor muscle strength in the operated knee. The difference regarding BMI is probably since AMVIC and NMVIC was combined as one variable in the above-mentioned meta-analysis of knee extensor muscle strength. It seems reasonable that higher BMI would be associated with higher AMVIC.

### NMVIC in the operated knee

Preoperative factors associated with higher normalised knee extensor muscle strength (NMVIC) on the operated side from baseline up to 52 weeks in our final model were: male sex, lower BMI and better 6MWT and 30CST performance, whereas age was not significant. Our findings support recommendations for people with knee OA to maintain physical activity and exercise over time, and to strive for normal weight, as outlined in the Swedish nationwide programme “Better management of patients with OA” (BOA) [42].

An interesting interpretation of our findings is that modifiable factors (BMI and physical performance) seem to be of more importance than the non-modifiable factor, age. Better preoperative physical function can reasonably be assumed to indicate a more advantageous starting position, which is supported by previous research [43]. Consistent with our findings following TKA, higher BMI has been associated with a greater initial decrease in NMVIC [26] and slower initial recovery in NMVIC [44]. In contrast to our findings, the results of a meta-regression analysis [26] found that age was a positive predictor, sex ratio (males/females) a negative predictor and BMI was not a significant predictor at the 6- and 12-month follow-ups. These conflicting findings regarding the association between preoperative factors and changes in NMVIC following TKA may be attributed to methodological differences, such as different statistical methods, choice of covariates investigated and the operationalisation of NMVIC (i.e. strength in relation to body weight versus the aggregate of strength in relation to body weight and in relation to BMI). Aggregating NMVIC in relation to body weight and BMI is problematic given that higher body weight does not necessarily also mean higher BMI, as the latter is also dependant on height. In general [21] and following TKA [24, 26], there are various methods used to normalise strength in relation to body composition (e.g. weight, length, BMI), as well as different methods to measure strength (e.g. isometric or isokinetic and as force or torque), which taken together or separately make the comparison of results difficult. There is a need to further clarify the advantages and disadvantages of different methods so that we can better guide researchers and clinicians in selecting the appropriate method.

It should be noted that in our results, higher BMI was a negative predictor for NMVIC and a positive predictor for AMVIC. The impact on NMVIC is of more relevance, as NMVIC has a better correlation with functional tests in general [21], as well as following TKA [15]. Thus, this study supports previous recommendations that obese patients (BMI >30) should be encouraged to lose weight before TKA to improve functional outcomes [45, 46]. In addition, weight loss is important as it reduces the risk for postoperative deep infections and arthroplasty revision [47].

### 6MWT, 30CST, KOOS

In this study, all patient-reported symptoms, pain, ADL and knee-related QoL (all KOOS-subscores) increased at all time points. Interestingly, the greatest increase was during the initial 12 weeks, when knee extensor AMVIC and NMVIC also decreased. Moreover, 30 CST was unchanged, and walking ability was increased during the same time. From 12 weeks to 52 weeks, significant improvements were seen for both walking ability and 30CST. This initial increase in 6MWT and patient-reported outcomes is probably related to pain reduction. KOOS-scores after TKA have previously also been reported to be improved at 1, 3 and 6 months after TKA [48]. For 6MWT, improvements have been reported at 3 and 6 months [48], as well as recovery to preoperative levels without significant improvements at 3 and 6 months [49]. At 3–6 months, it is quite common in clinical practice to encounter a satisfied patient with acceptable knee flexion (around 110°) for ADL [49, 50], pain reduction and improved walking ability. This patient should probably still be encouraged to continue to exercise, as the findings of this study and other studies demonstrate that functional performance is still impaired [9, 10, 23, 48, 49].

### Limitations and methodological considerations

There are limitations to this study that may affect the generalisability of the findings and should be considered when interpreting the results. These include a potential risk of type II error in the subgroup analysis involving women, selection and attrition bias, and the lack of detailed data on physiotherapy treatment beyond 6 weeks postoperatively. Among the study’s strengths is the use of a linear mixed effects model and the combination of baseline covariates assessed. Additionally, the use of hand-held dynamometry to assess knee extensor strength provides results that are readily applicable for comparison in clinical practice, as this method is both practical and accessible in routine care.

The total dropout rate was 25% (men, 23%; women, 27%). As a result, 16 women completed the study. As the power calculation for this study indicated that 22 subjects per group was needed, there is a risk for type II error in the subgroup analysis with women, meaning that potential significances have been missed. For instance, estimated marginal means in our data have quite large CIs when subgrouped by sex. As performance on tests of physical function varies between men and women and recovery may also vary between men and women, it would have been beneficial to consider them as separate groups when planning the study. The lack of detailed data on physiotherapy treatment beyond 6 weeks postoperatively may be considered as an important limitation. It is logical to assume that the load management strategy is important for overall knee function and specifically for knee extensor muscle strength. However, different rehabilitation strategies show comparable results for knee function after TKA [3–7].

Post hoc analyses indicate possible attrition bias, as linear mixed model results for AMVIC differed somewhat as age and the statistical interaction between time and sex were no longer significant. Additionally, dropouts had more pain, worse self-reported ADL and lower 30CST performance at baseline. It is also likely that the study attracted people with better functional performance than the average of the 82 subjects who declined to participate. When considering the group as a whole, performance on strength testing, 6MWT and 30CST is likely to be somewhat overestimated due to the lower proportion of women (39%) than in Swedish national data, where women represent 56% of all TKA patients [13]. Also, only 1 participant (2%) reported overall dissatisfaction at the 52-week follow-up, which is considerably lower than 8% in Swedish national data [13].

Using a linear mixed effect model is a strength of this study as it enabled us to assess and adjust for possible baseline covariates, to accommodate missing data points (which frequently occurs in longitudinal studies) and to model nonlinear individual characteristics [51]. Another strength is the inclusion of physical performance as a possible baseline covariate in addition to the more commonly assessed covariates (e.g. age, sex and BMI).

Regarding the approach to measuring knee extensor muscle strength, the results of our method (i.e. maximum voluntary isometric contraction using fixated hand-held dynamometry) were similar to findings in previous meta-analyses [24, 26] that included various methods (e.g. isokinetic devices, hand-held dynamometry, 1RM, torque, power, etc.) and can thus be seen as supporting previous statements regarding the reliability and validity of the method [27]. Moreover, the method is easy to apply in everyday clinical practice, especially compared to many of the other above-mentioned methods, which are more time-consuming, expensive and space occupying (i.e. isokinetic devices).

## Conclusions

Better preoperative physical function and lower BMI appear beneficial for postoperative recovery of normalised knee extensor muscle strength. Following total knee arthroplasty, patient-reported outcomes (symptoms, pain, ADL and knee-related QoL) as well as gait performance appear to improve more rapidly than knee extensor muscle strength and 30CST. The clinical implication of the study is that preoperative exercise and weight loss (if obese) may be advisable. Postoperatively, the clinical implication is that exercises aimed at improving knee extensor muscle strength may be advisable even if pain is alleviated and walking is improved.

## Data Availability

Data available on request due to privacy and ethical restrictions. The data that support the findings of this study are available on request from the corresponding author (PS). The data are not publicly available due to ethical and legal restrictions, as the data contain information that could compromise the privacy of research participants.

## Abbreviations

30CST: 30s chair stand test
6MWT: 6-min walk test
ADL: Activities of daily living
AMVIC: Absolute maximum voluntary isometric contraction
BMI: Body mass index
KOOS: Knee injury and osteoarthritis outcome score
MVIC: Maximum voluntary isometric contraction
N: Newtons
OA: Osteoarthritis
NMVIC: Normalised maximum voluntary isometric contraction
OARSI: Osteoarthritis research society international
PS: Per Sjöström (corresponding author)
QoL: Quality of life
ROM: Range of motion
SD: Standard deviation
TKA: Total knee arthroplasty

## References

[1] Nemes S, Rolfson O, W-Dahl A, Garellick G, Sundberg M, Kärrholm J, et al. Historical view and future demand for knee arthroplasty in Sweden. Acta Orthop. 2015;86(4):426–31. 25806653 10.3109/17453674.2015.1034608 PMC4513596

[2] OECD. Hip and knee replacement. Paris: OECD Publishing. 2019. Available from: https://www.oecd-ilibrary.org/content/component/2fc83b9a-en. Cited 2021 May 12.

[3] Dávila Castrodad IM, Recai TM, Abraham MM, Etcheson JI, Mohamed NS, Edalatpour A, et al. Rehabilitation protocols following total knee arthroplasty: a review of study designs and outcome measures. Ann Transl Med. 2019;7(Suppl 7):S255. 31728379 10.21037/atm.2019.08.15 PMC6829007

[4] Alrawashdeh W, Eschweiler J, Migliorini F, Mansy Y, Tingart M, Rath B. Effectiveness of total knee arthroplasty rehabilitation programmes: a systematic review and meta-analysis. J Rehabil Med. 2021;53(6):jrm00200–jrm. 33846757 10.2340/16501977-2827 PMC8814866

[5] Ginnetti JG, O’Connor MI, Chen AF, Myers TG. Total joint arthroplasty training (prehabilitation and rehabilitation) in lower extremity arthroplasty. JAAOS - J Am Acad Orthop Surg. 2022;30(11):e799-807. 35594512 10.5435/JAAOS-D-21-00247

[6] Jansson MM, Rantala A, Miettunen J, Puhto A-P, Pikkarainen M. The effects and safety of telerehabilitation in patients with lower-limb joint replacement: a systematic review and narrative synthesis. J Telemed Telecare. 2022;28(2):96–114. 32316837 10.1177/1357633X20917868

[7] Konnyu KJ, Thoma LM, Cao W, Aaron RK, Panagiotou OA, Bhuma MR, et al. Rehabilitation for total knee arthroplasty: a systematic review. Am J Phys Med Rehabil. 2023;102(1):19–33. 35302953 10.1097/PHM.0000000000002008 PMC9464796

[8] Canovas F, Dagneaux L. Quality of life after total knee arthroplasty. Orthop Traumatol Surg Res. 2018;104(1):S41-6. 29183821 10.1016/j.otsr.2017.04.017

[9] Capin JJ, Bade MJ, Jennings JM, Snyder-Mackler L, Stevens-Lapsley JE. Total knee arthroplasty assessments should include strength and performance-based functional tests to complement range-of-motion and patient-reported outcome measures. Phys Ther. 2022;102(6):1. 10.1093/ptj/pzac033 PMC9393064 35358318

[10] Mizner R, Stevens J, Snyder-Mackler L. Voluntary activation and decreased force production of the quadriceps femoris muscle after total knee arthroplasty. Phys Ther. 2003;83(4):359–65. 12665406

[11] Terwee CB, van der Slikke RMA, van Lummel RC, Benink RJ, Meijers WGH, de Vet HCW. Self-reported physical functioning was more influenced by pain than performance-based physical functioning in knee-osteoarthritis patients. J Clin Epidemiol. 2006;59(7):724–31. 16765276 10.1016/j.jclinepi.2005.11.019

[12] Berg U, Annette WD, Ola R, Emma N, Martin S, Nilsdotter A. Influence of fast-track programs on patient-reported outcomes in total hip and knee replacement (THR/TKR) at Swedish hospitals 2011–2015: an observational study including 51,169 THR and 8,393 TKR operations. Acta Orthop. 2020;91(3):306–12. 32106731 10.1080/17453674.2020.1733375 PMC8023888

[13] W-Dahl A, Kärrholm J, Rogmark C, Johansson O, Arani PI, Mohaddes M, Rolfson O. Annual report 2024: The Swedish arthroplasty register; 2025. Available from: https://registercentrum.blob.core.windows.net/sar/r/Swedish-Arthroplasty-Register-Annual-report-2024-ENG-CHsgLK06p.pdf.

[14] Mahdi A, Svantesson M, Wretenberg P, Hälleberg-Nyman M. Patients’ experiences of discontentment one year after total knee arthroplasty- a qualitative study. BMC Musculoskelet Disord. 2020;21(1):29. 31937282 10.1186/s12891-020-3041-y PMC6961288

[15] Marmon AR, Milcarek BI, Snyder-Mackler L. Associations between knee extensor power and functional performance in patients after total knee arthroplasty and normal controls without knee pain. Int J Sports Phys Ther. 2014;9(2):168. 24790778 PMC4004122

[16] Mizner RL, Petterson SC, Snyder-Mackler L. Quadriceps strength and the time course of functional recovery after total knee arthroplasty. J Orthop Sports Phys Ther. 2005;35(7):424–36. 16108583 10.2519/jospt.2005.35.7.424

[17] Pozzi F, Snyder-Mackler L, Zeni J. Physical exercise after knee arthroplasty: a systematic review of controlled trials. Eur J Phys Rehabil Med. 2013;49(6):877–92. 24172642 PMC4131551

[18] Goodpaster BH, Park SW, Harris TB, Kritchevsky SB, Nevitt M, Schwartz AV, et al. The loss of skeletal muscle strength, mass, and quality in older adults: the health, aging and body composition study. J Gerontol A Biol Sci Med Sci. 2006;61(10):1059–64. 10.1093/gerona/61.10.1059 17077199

[19] Hughes VA, Frontera WR, Wood M, Evans WJ, Dallal GE, Roubenoff R, et al. Longitudinal muscle strength changes in older adults: influence of muscle mass, physical activity, and health. J Gerontol A Biol Sci Med Sci. 2001;56(5):B209-17. 11320101 10.1093/gerona/56.5.b209

[20] Bohannon RW. Reference values for extremity muscle strength obtained by hand-held dynamometry from adults aged 20 to 79 years. Arch Phys Med Rehabil. 1997;78(1):26–32. 9014953 10.1016/s0003-9993(97)90005-8

[21] Jaric S. Muscle strength testing: use of normalisation for body size. Sports Med. 2002;32(10):615–31. 12141882 10.2165/00007256-200232100-00002

[22] Vahtrik D, Gapeyeva H, Ereline J, Pääsuke M. Relationship between leg extensor muscle strength and knee joint loading during gait before and after total knee arthroplasty. Knee. 2014;21(1):216–20. 23721904 10.1016/j.knee.2013.05.002

[23] Valtonen A, Pöyhönen T, Heinonen A, Sipilä S. Muscle deficits persist after unilateral knee replacement and have implications for rehabilitation. Phys Ther. 2009;89(10):1072. 19713269 10.2522/ptj.20070295

[24] Singla R, Niederer D, Franz A, Happ K, Zilkens C, Wahl P, et al. The course of knee extensor strength after total knee arthroplasty: a systematic review with meta-analysis and -regression. Arch Orthop Trauma Surg. 2023. 10.1007/s00402-022-04750-5. 36637491 10.1007/s00402-022-04750-5 PMC10374784

[25] Ali A, Lindstrand A, Nilsdotter A, Sundberg M. Similar patient-reported outcomes and performance after total knee arthroplasty with or without patellar resurfacing: a randomized study of 74 patients with 6 years of follow-up. Acta Orthop. 2016;87(3):274–9. 27212102 10.3109/17453674.2016.1170548 PMC4900081

[26] Paravlic AH, Meulenberg CJ, Drole K. The time course of quadriceps strength recovery after total knee arthroplasty is influenced by body mass index, sex, and age of patients: systematic review and meta-analysis. Front Med. 2022;9:865412. 10.3389/fmed.2022.865412 PMC9174520 35692543

[27] Gagnon D, Nadeau S, Gravel D, Robert J, Bélanger D, Hilsenrath M. Reliability and validity of static knee strength measurements obtained with a chair-fixed dynamometer in subjects with hip or knee arthroplasty. Arch Phys Med Rehabil. 2005;86(10):1998–2008. 16213245 10.1016/j.apmr.2005.04.013

[28] Crosbie J, Naylor JM, Harmer AR. Six minute walk distance or stair negotiation? Choice of activity assessment following total knee replacement. Physiother Res Int. 2010;15(1):35–41. 20052684 10.1002/pri.453

[29] Jakobsen T, Kehlet H, Bandholm T. Reliability of the 6-min walk test after total knee arthroplasty. Knee Surg Sports Traumatol Arthrosc. 2013;21(11):2625–8. 22644072 10.1007/s00167-012-2054-y

[30] Kennedy DM, Stratford PW, Wessel J, Gollish JD, Penney D. Assessing stability and change of four performance measures: a longitudinal study evaluating outcome following total hip and knee arthroplasty. BMC Musculoskelet Disord. 2005;6(1):3. 15679884 10.1186/1471-2474-6-3 PMC549207

[31] Dobson F, Hinman RS, Roos EM, Abbott JH, Stratford P, Davis AM, et al. OARSI recommended performance-based tests to assess physical function in people diagnosed with hip or knee osteoarthritis. Osteoarthritis Cartilage. 2013;21(8):1042–52. 23680877 10.1016/j.joca.2013.05.002

[32] Gill S, McBurney H. Reliability of performance-based measures in people awaiting joint replacement surgery of the hip or knee. Physiother Res Int. 2008;13(3):141–52. 18697226 10.1002/pri.411

[33] Jones CJ, Rikli RE, Beam WC. A 30-s chair-stand test as a measure of lower body strength in community-residing older adults. Res Q Exerc Sport. 1999;70(2):113–9. 10380242 10.1080/02701367.1999.10608028

[34] Unver B, Kalkan S, Yuksel E, Kahraman T, Karatosun V. Reliability of the 50-foot walk test and 30-sec chair stand test in total knee arthroplasty. Acta Ortop Bras. 2015;23(4):184. 26327798 10.1590/1413-78522015230401018 PMC4544525

[35] Roos EM. The 2012 users’ guide to: Knee injury osteoarthritis outcome score KOOS 2012. Available from: https://koos.nu/KOOSusersguide2012_RC.pdf. Updated Jan 20252025-06-24 .

[36] Collins NJ, Misra D, Felson DT, Crossley KM, Roos EM. Measures of knee function: international knee documentation committee (IKDC) subjective knee evaluation form, knee injury and osteoarthritis outcome score (KOOS), knee injury and osteoarthritis outcome score physical function short form (KOOS-PS), knee outcome survey activities of daily living scale (KOS‐ADL). Lysholm knee scoring scale, Oxford knee score (OKS), Western Ontario and McMaster universities osteoarthritis index (WOMAC), activity rating scale (ARS), and Tegner activity score. TAS. Arthritis Care Res. 2011;63(S11):S208–S28. 10.1002/acr.20632 PMC4336550 22588746

[37] Roos EM, Toksvig-Larsen S. Knee injury and osteoarthritis outcome score (KOOS) - validation and comparison to the WOMAC in total knee replacement. Health Qual Life Outcomes. 2003;1:17. 12801417 10.1186/1477-7525-1-17 PMC161802

[38] Olsson SJG, Ekblom Ö, Andersson E, Börjesson M, Kallings LV. Categorical answer modes provide superior validity to open answers when asking for level of physical activity: a cross-sectional study. Scand J Public Health. 2016;44(1):70–6. 26392418 10.1177/1403494815602830

[39] Kahlenberg C, Nwachukwu B, McLawhorn A, Cross M, Cornell C, Padgett D. Patient satisfaction after total knee replacement: a systematic review. HSS Journal ®. 2018;14(2):192–201. 29983663 10.1007/s11420-018-9614-8 PMC6031540

[40] Tabachnick BG. In: Fidell LS, editor. Using multivariate statistics. 6., pearson new international edition ed. Harlow: Pearson; 2014.

[41] Keating JL, Matyas TA. The influence of subject and test design on dynamometric measurements of extremity muscles. Phys Ther. 1996;76(8):866–89. 8710966 10.1093/ptj/76.8.866a

[42] Thorstensson CA, Garellick G, Rystedt H, Dahlberg LE. Better management of patients with osteoarthritis: development and nationwide implementation of an evidence-based supported osteoarthritis self-management programme. Musculoskelet Care. 2015;13(2):67–75. 10.1002/msc.1085 25345913

[43] Topp R, Swank AM, Quesada PM, Nyland J, Malkani A. The effect of prehabilitation exercise on strength and functioning after total knee arthroplasty. Pm&r. 2009;1(8):729–35. 19695525 10.1016/j.pmrj.2009.06.003

[44] Pua YH, Seah FJT, Seet FJH, Tan JWM, Liaw JSC, Chong HC. Sex differences and impact of body mass index on the time course of knee range of motion, knee strength, and gait speed after total knee arthroplasty. Arthritis Care Res. 2015;67(10):1397–405. 10.1002/acr.22584 25776869

[45] Si H-b, Zeng Y, Shen B, Yang J, Zhou Z-k, Kang P-d, Pei F-x. The influence of body mass index on the outcomes of primary total knee arthroplasty. Knee Surg Sports Traumatol Arthroscopy: Official J ESSKA. 2015;23(6):1824–32. 10.1007/s00167-014-3301-1 25217315

[46] Xu S, Chen JY, Lo NN, Chia SL, Tay DKJ, Pang HN, et al. The influence of obesity on functional outcome and quality of life after total knee arthroplasty. Bone Joint J. 2018;100–B(5):579–83. 29701098 10.1302/0301-620X.100B5.BJJ-2017-1263.R1

[47] Kerkhoffs GMMJ, Servien E, Dunn W, Dahm D, Bramer JAM, Haverkamp D. The influence of obesity on the complication rate and outcome of total knee arthroplasty: a meta-analysis and systematic literature review. J Bone Joint Surg Am. 2012;94(20):1839–44. 23079875 10.2106/JBJS.K.00820 PMC3489068

[48] Stevens-Lapsley JE, Schenkman ML, Dayton MR. Comparison of Self-Reported knee injury and osteoarthritis outcome score to performance measures in patients after total knee arthroplasty. PM&R. 2011;3(6):541–9. 21665167 10.1016/j.pmrj.2011.03.002

[49] Bade MJ, Kohrt WM, Stevens-Lapsley JE. Outcomes before and after total knee arthroplasty compared to healthy adults. J Orthop Sports Phys Therapy. 2010;40(9):559–67. 10.2519/jospt.2010.3317 PMC3164265 20710093

[50] Kittelson AJ, Elings J, Colborn K, Hoogeboom TJ, Christensen JC, van Meeteren NLU, et al. Reference chart for knee flexion following total knee arthroplasty: a novel tool for monitoring postoperative recovery. BMC Musculoskelet Disord. 2020;21(1):482. 32698900 10.1186/s12891-020-03493-x PMC7376933

[51] Krueger C, Tian L. A comparison of the general linear mixed model and repeated measures ANOVA using a dataset with multiple missing data points. Biol Res Nurs. 2004;6(2):151–7. 15388912 10.1177/1099800404267682

